# Bonding to Dentin Contaminated with Ceramic-Repair Primers/Etchants

**DOI:** 10.3290/j.jad.c_2336

**Published:** 2025-11-13

**Authors:** Apinya Limvisitsakul, Thawatchai Likhitthaworn, Saowaros Kaophun, Bart Van Meerbeek, Pong Pongprueksa

**Affiliations:** a Apinya Limvisitsakul Lecturer, Mahidol University, Faculty of Dentistry, Department of Operative Dentistry and Endodontics, 6 Yothi Street, Ratchathewi, Bangkok, 10400, Thailand. Experimental design, wrote the manuscript, proofread the manuscript, performed statistical evaluation, and contributed substantially to the discussion.; b Thawatchai Likhitthaworn Postgraduate student, Mahidol University, Faculty of Dentistry, Department of Operative Dentistry and Endodontics, 6 Yothi Street, Ratchathewi, Bangkok, 10400, Thailand. Hypothesis, experimental design, performed the experiments in partial fulfillment of requirements for a degree, wrote the manuscript, and performed part of the experiment.; c Saowaros Kaophun Research Technical Officer, Mahidol University, Faculty of Dentistry, Dental Biomaterial Analysis and Research Center, 6 Yothi Street, Ratchathewi, Bangkok, 10400, Thailand. Consulted on the methodology and performed part of the experiments.; d Bart Van Meerbeek Full Professor, KU Leuven, Department of Oral Health Sciences, BIOMAT & UZ Leuven, Dentistry, Kapucijnenvoer 7, Blok a – Box 7001, BE-3000 Leuven, Belgium. Proofread the manuscript and contributed to the discussion.; e Pong Pongprueksa Associate Professor, Mahidol University, Faculty of Dentistry, Department of Operative Dentistry and Endodontics, 6 Yothi Street, Ratchathewi, Bangkok, 10400, Thailand. Idea, supervision, hypothesis, experimental design, proofread the manuscript, performed statistical evaluation, and contributed to the discussion.

**Keywords:** ammonium polyfluoride, calcium fluoride, hydrofluoric acid, Monobond Etch & Prime, universal adhesive

## Abstract

**Purpose:**

To evaluate bonding to dentin contaminated with primers/etchants used for adjacent ceramic repair.

**Materials and Methods:**

Mid-coronal dentin of sound human third molars was exposed and allocated to 10 experimental groups. The universal adhesive (UA) Single Bond Universal (“SBU,” 3M Oral Care), applied either in etch-and-rinse (E&R) or self-etch (SE) bonding mode, and the considered gold-standard SE adhesive Clearfil SE Bond X (“CSE,” Kuraray Noritake) were bonded to dentin contaminated with either Monobond Etch & Prime (“MEP,” Ivoclar) or IPS Ceramic Etching Gel (“HF,” Ivoclar) following 10 scenarios: phosphoric acid (PA)+SBU_E&R_ (uncontaminated E&R UA control), HF+PA+SBU_E&R_, MEP+PA+SBU_E&R_, PA+MEP+SBU_E&R_, SBU_SE_ (uncontaminated SE UA control), HF+SBU_SE_, MEP+SBU_SE_, CSE_SE_ (uncontaminated SE control), HF+CSE_SE_, MEP+CSE_SE_. Upon adhesive and composite application, the specimens were stored in artificial saliva at 37°C. After 1 week, all specimens were sectioned into resin-bonded dentin sticks, which were randomly distributed over two groups to be subjected to a microtensile bond-strength test immediately at 1 week or upon aging by storage in artificial saliva for 6 months. Statistics involved linear mixed-effects modeling with Bonferroni correction (P <0.05).

**Results:**

E&R bonding to dentin contaminated with MEP or HF did not significantly differ from bonding to non-contaminated dentin (controls). However, SE bonding to MEP- and HF-contaminated dentin was significantly less effective than to non-contaminated dentin (controls). Aging for 6 months did not reduce E&R bonding as compared to the 1-week data, while SE bonding was significantly less effective upon 6-month aging. E&R bonding was affected more when dentin was contaminated with MEP before phosphoric acid (PA) etching than when dentin was contaminated with MEP after PA etching.

**Conclusions:**

Dentin contamination with MEP and HF impacted self-etch (SE) bonding but not etch&rinse (E&R) bonding.

Fracture and chipping are commonly regarded as the main causes of failure of ceramic restorations.^2^ Fractured ceramic restorations are often better intraorally repaired with resin composite than replaced, depending on the extent of damage to the residual tooth structure and restoration.^6,7^ Repairing ceramics with composite resin requires micromechanical and chemical bonding procedures. To enhance micromechanical interlocking, acid etching, sandblasting, diamond-bur cutting, and laser etching can be used. Chemical binding can be realized using silanes and adhesives, which are of primary importance for repair/bond durability.^22^

Repairing glass-ceramics specifically involves etching with hydrofluoric acid (HF) in a concentration ranging between 2.5 and 10%.^11^ This caustic etching agent should be removed through thorough water rinsing, followed by complete air-drying. The HF-etched ceramic surface is next silanized using a dedicated ceramic or universal restoration primer, containing silane and other functional monomers, followed by the application of an adhesive in either etch-and-rinse (E&R) or self-etch (SE) bonding mode, enabling bonding to adjacent tooth structure as well. After separate light-curing of the adhesive, the restoration defect is repaired with resin composite, commonly incrementally layered.^13,16^

When repairing ceramic restorations, adjacent dentin cannot always be avoided from being exposed to the ceramic-repair primers/etchants, by which the resultant bond to dentin may be adversely affected. Dentin specimens contaminated with 3 and 9.6% HF appeared significantly less bonding receptive than non-contaminated control dentin.^12^ Hence, ceramic repair with composite can be complicated when the fracture also involves enamel and/or dentin exposure. The agents used to repair ceramics can alter the physical and chemical properties of the exposed tooth structure, potentially impacting bonding receptiveness.^18^

The single-component self-etching ceramic primer, being marketed as Monobond Etch & Prime (“MEP”; Ivoclar, Schaan, Liechtenstein), combines an etching agent based on ammonium polyfluoride with a methacrylate silane, which is much less aggressive/caustic than HF and consequently attractive for intraoral ceramic repairs. The one-step procedure involves simultaneously etching and silanizing the glass-ceramic surface, upon which the adhesive can be applied. The two functional components of this self-etching ceramic primer are bis(triethoxysilyl)ethane as a silane coupling agent and tetrabutylammonium dihydrogen trifluoride as a (self-)etching agent. The latter etching agent is a safer derivative of hydrofluoric acid for intraoral application, as confirmed by the manufacturer Ivoclar. Hydrofluoric acid is a hazardous agent that requires careful use to avoid damage to the skin, lips, mucosa, and gingiva.^4,8,17,25^ The simplified one-step application of MEP is also considered less technique-sensitive. Some previous studies found no significant difference in bond strength and durability between etching glass-ceramic with conventional HF, followed by application of a separate silane primer, and the single use of the self-etching ceramic primer MEP.^23,25^

The objective of this study was to evaluate the effect of dentin surfaces contaminated with ceramic priming/etching agents on the bonding performance of two adhesives, hereby employing a microtensile bond-strength (µTBS) approach. The findings from this parameter-controlled experiment can be used to provide guidelines to clinicians in selecting the appropriate materials and procedures when intraorally repairing ceramics. The null hypothesis tested in this study was that there would not be any significant difference in µTBS (1) among the different experimental scenarios and (2) between the 1-week immediate and 6-month aged µTBS.

## MATERIALS AND METHODS

The study protocol was approved by the Ethics Committee (COE.No.MU-DT/PY-IRB 2020/002.0602). Ninety-four non-carious human third molars were stored in 0.1% thymol solution at 4°C. The storage solution of extracted teeth was changed to normal saline 24 h before use.

### Microtensile Bond Strength (*µ*TBS)

Eighty teeth were sectioned horizontally, removing 1/3 of the occlusal part to expose mid-coronal dentin perpendicular to the long axis of the tooth using a low-speed cutting machine (IsoMet; Buehler, Lake Bluff, IL, USA) with a water-cooled diamond blade (M1D10; Struers, Ballerup, Denmark). The dentin surface was ground in a linear motion with 600-grit silicone-carbide (SiC) paper (CarbiMet; Buehler) under running water for 30 strokes to produce a standardized smear layer on the dentin surface. All tooth specimens were distributed over 10 experimental groups and processed according to the experimental scenarios detailed underneath, as well as mentioned in Table 1 along with the composition of all materials employed in this study.

**Table 1:** Material composition and application method used in this study

Materials	Composition^1^	Application method
Monobond Etch & Prime (MEP) (lot-no. Y27773) *Ivoclar, Schaan, Liechtenstein*	tetrabutylammonium dihydrogen trifluoride (≤10 wt%), methacrylated phosphoric acid ester (3–10 wt%), butanol (10–25 wt%), bis(triethoxysilyl)ethane (1–2.5 wt%), 3-(trimethoxysilyl)propyl methacrylate, water	Application of MEP for 20 s, allowed to react with the surface for 40 s, rinsed with water, followed by 10-s gently air-drying.
IPS Ceramic Etching gel (HF) (lot-no. Y06707) *Ivoclar*	hydrofluoric acid (<5 wt%)	Application of HF gel on the surface for 20 s, followed by rinsing under running water, and gently air-drying
Single Bond Universal (SBU) (lot-no. 90913B) *3M Oral Care and now Solventum, Seefeld, Germany*	Bis-GMA (15–25 wt%), HEMA (15–25 wt%), 10-MDP (5–15 wt%), ethanol (10–15 wt%), water (10–15 wt%), silane-treated silica (5–10 wt%), copolymer of acrylic and itaconic acid (1–5 wt%), methyl ethyl ketone (<0.5 wt%), CQ (˷2 wt%), EDMAB (<2 wt%)	Application of phosphoric acid (PA) gel etchant on dentin for 15 s, followed by rinsing for 15 s, and gently air-drying; rubbed application of UA for 20 s, followed by 5-s gently air-drying, and 10-s light curing (solely the latter step in case of SE bonding mode).
Clearfil SE Bond X^2^ (CSE) (Primer lot-no. 1Q0283, Bond lot-no. 1X0454) Kuraray Noritake, Tokyo, Japan	*Primer:* HEMA (20–40 wt%), 10-MDP, hydrophilic aliphatic dimethacrylate, CQ, water *Bond: *Bis-GMA (25–45 wt%), HEMA (20–40 wt%), 10-MDP, hydrophobic aliphatic dimethacrylate, colloidal silica, CQ	Rubbed application of primer for 20 s, followed by 5-s mild air-drying; application of “Bond,” followed by gently air-drying and 10-s light curing.
Filtek Z250 (lot-no. NA59091) 3M Oral Care/Solventum	Bis-GMA (1-10wt%), TEGDMA (<5 wt%), UDMA (1–10 wt%), Bis-EMA(6) (1–10wt%), silane treated ceramic filler (75–85 wt%)	Incrementally layered with 1.5–2-mm layers and light-cured for 20 s.
^1^ According to technical information provided by the respective manufacturers with the following abbreviations: Bis-EMA(6): bisphenol A ethoxylateddimethacrylate; Bis-GMA: bisphenol A-glycidyl dimethacrylate; CQ, camphorquinone; EDMAB: ethyl 4-(dimethylamino)benzoate; HEMA: 2-hydroxyethyl methacrylate; TEGDMA: triethylene glycol dimethacrylate; UDMA: diurethane dimethacrylate; 10-MDP: 10-met.hacryloyloxydecyl dihydrogen phosphate; ^2^ Elsewhere marketed by Kuraray Noritake as Clearfil SE Bond.

#### Group 1

PA+SBU_E&R_ (uncontaminated E&R UA control) – The universal adhesive (UA) Single Bond Universal (“SBU”; 3M Oral Care, currently Solventum, Seefeld, Germany) was applied in E&R bonding mode, strictly following the manufacturer’s instructions. Upon smear-layer preparation, dentin was etched with 35% phosphoric acid (“PA”; Scotchbond Universal Etchant, 3M Oral Care) for 15 s and rinsed with water spray for 10 s, upon which excess water was removed using a cotton pellet to achieve moist dentin. The adhesive was applied and rubbed for 20 s onto the entire dentin surface, followed by 5 s of gentle air-drying until the adhesive no longer moved. The adhesive was light-cured for 10 s using the LED light-curing unit Bluephase N (Ivoclar) set at a “high power” mode that emitted a light intensity of approximately 1,000 mW/cm^2^ as measured by a radiometer (Bluephase Meter II; Ivoclar).

#### Group 2

HF+PA+SBU_E&R_ – Dentin was intentionally etched/contaminated with 5% hydrofluoric acid (“HF”; IPS Ceramic etching gel; Ivoclar) for 20 s, rinsed with water spray and dried with strong air for 10 s. SBU was applied in E&R bonding mode, as detailed for Group 1.

#### Group 3

MEP+PA+SBU_E&R_ – Dentin was intentionally (self-)etched/contaminated with the self-etching ceramic primer Monobond Etch & Prime (“MEP”; Ivoclar) by agitating the surface for 20 s using a microbrush and then allowed it to react for another 40 s, according to the application instructions of MEP. The primer was rinsed with a water spray and dried with strong air for 10 s. SBU was again applied in E&R bonding mode, as detailed for Group 1.

#### Group 4

PA+MEP+SBU_E&R_ – SBU was applied in E&R bonding mode following the same procedure as Group 3 but with MEP applied after PA etching.

#### Group 5

SBU_SE_ (uncontaminated SE UA control) – SBU was applied in SE bonding mode. The adhesive was rubbed onto dentin for 20 s, followed by 5 s of gentle air-drying until the adhesive no longer moved. The adhesive was light-cured for 10 s.

#### Group 6

HF+SBU_SE_ – Dentin was intentionally etched/contaminated with 5% HF before applying SBU in SE bonding mode, as detailed for Group 5.

#### Group 7

MEP+SBU_SE_ – Dentin was intentionally (self-)etched/contaminated with MEP, as described in detail for Group 3, upon which SBU was applied in SE bonding mode using the same procedure as described for Group 5.

#### Group 8

CSE_SE_ (uncontaminated SE control) – The two-step self-etch (SE) adhesive (“CSE”; Clearfil SE Bond X, Kuraray Noritake, Tokyo, Japan; marketed by Kuraray Noritake as Clearfil SE Bond in other parts of the world) was applied in SE bonding mode, by first rubbing the primer onto dentin for 20 s, followed by 5 s gentle air-drying until the primer no longer moved. The adhesive was then applied, gently air-dried, and light-cured for 10 s.

#### Group 9

HF+CSE_SE_ – Dentin was intentionally etched/contaminated with 5% HF before applying CSE using the same procedure as detailed for Group 8.

#### Group 10

MEP+CSE_SE_ – Dentin was intentionally (self-)etched/contaminated with MEP, as described in detail for Group 3, upon which CSE was applied using the same procedure as in Group 8.

Upon adhesive application, all specimens were built up with composite (Filtek Z250, 3M Oral Care) in successive 2-mm incremental layers to reach a height of 5 mm. Each layer was separately light-cured for 20 s using the LED light-curing unit Bluephase N (Ivoclar). The specimens were next kept in artificial saliva at 37°C for 1 week. The artificial saliva was prepared by mixing NaCl (0.798 g), KCl (1.2 g), CaCl_2_ (0.147 g), KH_2_PO_4_ (0.272 g), MgCl_2_.6H_2_O (0.093 g), and deionized water (990 ml) to form 1,000 ml of solution. NaOH with a concentration of 1 mol/L was added to adjust the pH to 7 under the pH meter (Benchtop pH, Thermo Fisher Scientific, MA, USA).1

The teeth were sectioned perpendicularly through the interface using an automatic precision cutting machine (Accutom-50, Struers) with a water-cooled diamond blade (M1D10, Struers) employed using a 1500-rpm wheel speed to produce rectangular 1×1 mm sticks. The central sticks were used for µTBS testing. Half of the sticks were tested “immediately” upon sectioning of the micro-specimens at 1 week, while the other half were “aged” by storage in artificial saliva for 6 months prior to being subjected to µTBS testing. Each stick was attached to a modified notched Ciucchi’s jig^19^ using cyanoacrylate glue (Model Repair II Blue; Dentsply-Sankin, Tochigi, Japan) and stressed at a crosshead speed of 1 mm/min until failure in the universal testing machine (LRX Lloyd, Hampshire, UK) to determine the µTBS. Per tooth, the mean µTBS in MPa recorded for the sticks originating from that specific tooth was calculated, serving as the tooth-representative µTBS. Premature stick failures prior to testing were assigned a value of zero MPa and included in the statistical analysis.

The mode of failure was determined using stereomicroscopy (SMZ-2T; Nikon, Tokyo, Japan) at 40× magnification. The failure mode was classified as either “adhesive,” “mixed,” or “cohesive” failure.^21^

### Surface Morphology

Fourteen additional human third molars without caries were stored in a 0.1% thymol solution at 4°C. Prior to further processing, the storage solution was replaced with normal saline and the teeth were left to soak for 24 h. Each tooth was sectioned horizontally at the coronal 1/3 of the crown using a low-speed diamond saw (M1D10; Struers) to expose flat mid-coronal dentin, as well as 1 mm below the cementoenamel junction by a second cut, producing dentin discs. The exposed dentin surface of each disc was ground with 600-grit SiC paper (CarbiMet; Buehler) under running water in a linear motion for 30 s to ensure standardized smear-layer preparation.

The dentin discs were then prepared for a split-tooth design. A 150-μm ultrathin diamond blade (M1D08; Struers; feed speed of 0.015 mm/s, wheel speed of 1,500 rpm) was utilized to cut a thin and shallow groove into the exposed dentin surface under water lubrication. The specimens were randomly assigned to seven experimental groups (n = 2/group), in which dentin was conditioned as follows: (1) PA, (2) PA+MEP, (3) PA+HF, (4) MEP, (5) HF, (6) MEP+PA, and (7) HF+PA. The surface primers/etchants were applied to half of the occlusal surface, while the other half remained non-primed/etched, hereby serving as a control.

Following specimen cleaning in an ultrasonic bath for 10 min in distilled water, the dentin surface was gently dried with laboratory tissue paper (Kimwipes, Kimtech, Tainan City, Taiwan). The cleaned specimens were dehydrated in a graded series of ethanol (immersed in 25% ethanol for 20 min, 50% ethanol for 20 min, 75% ethanol for 20 min, 95% ethanol for 30 min, and finally 100% ethanol for 60 min). The specimens were then mounted on aluminum stubs and coated with palladium using a sputter coater (SC7620 Sputter coater, Quorum Technologies, Kent, UK) for morphologic surface observation using scanning electron microscopy (SEM; JSM-6610LV; JEOL, Tokyo, Japan) and chemical element analysis using energy dispersive X-ray spectroscopy (EDS).

### Statistical Analysis

A linear mixed-effects model created with R Foundation for Statistical Computing (Vienna, Austria) was used to statistically analyze the recorded μTBSs of the adhesives to the differently primed/etched dentin surfaces. Differences were considered significant when P <0.05. The fixed factors included in the analysis were the “experimental group” and “aging.” Post-hoc multiple comparisons were analyzed using a Bonferroni adjustment.

## RESULTS

Statistical analysis revealed that the variables “experimental group” and “aging” significantly contributed to the statistical model. Additionally, there was a statistically significant interaction between both variables, indicating that µTBS was impacted differently by the various experimental group conditions and aging (Table 2).

**Table 2:** Statistical analysis of the fixed variables and interaction for the LME model

	numDF	denDF	F value	P value
Experimental group	9	152	62.2212	<0.0001*
Ageing	1	152	15.6413	0.0001*
Experimental group/ageing interaction	9	152	3.6273	0.0003*
*Statistically significant.

**Fig 1:**
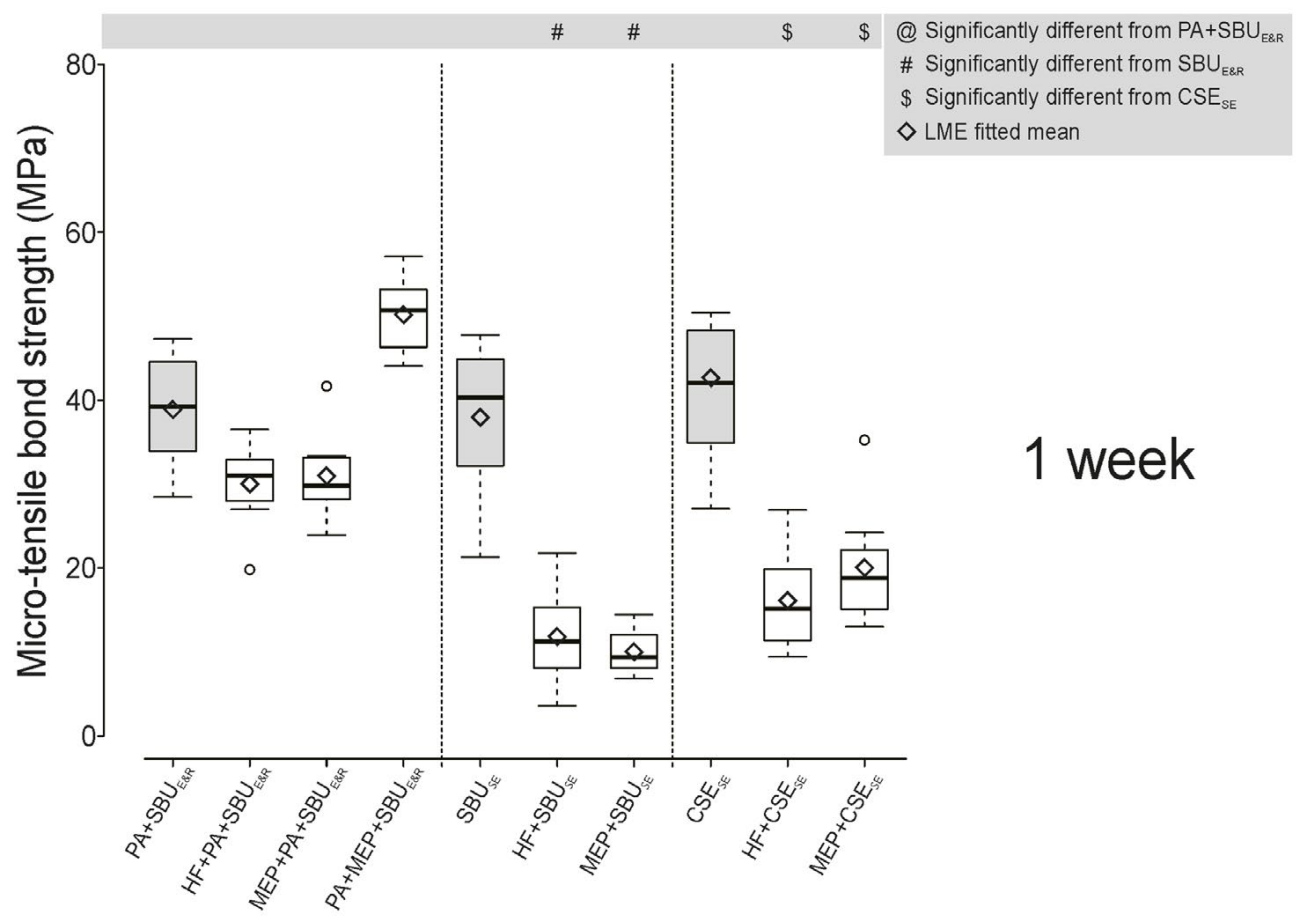
Box plots representing the 1-week micro-tensile bond strength (µTBS in MPa) of the universal adhesive Single Bond Universal (“SBU”; 3M Oral Care/Solventum), applied in etch-and-rinse (E&R) and self-etch (SE) bonding mode, and the considered gold-standard SE adhesive Clearfil SE Bond X (“CSE”; Kuraray Noritake) to dentin non-contaminated (SBU_E&R_, SBU_SE_, and CSE_SE_ controls) and contaminated with either hydrofluoric acid (HF) or the self-etching ceramic primer Monobond Etch & Prime (“MEP”; Ivoclar). The box represents the data spreading between the first and third quartile, with the whiskey denoting minimum and maximum. Black diamonds represent the mean µTBS with the horizontal line referring to the median. Statistically significant differences (P <0.05) are indicated by specific symbols in the figure legend.

**Fig 2:**
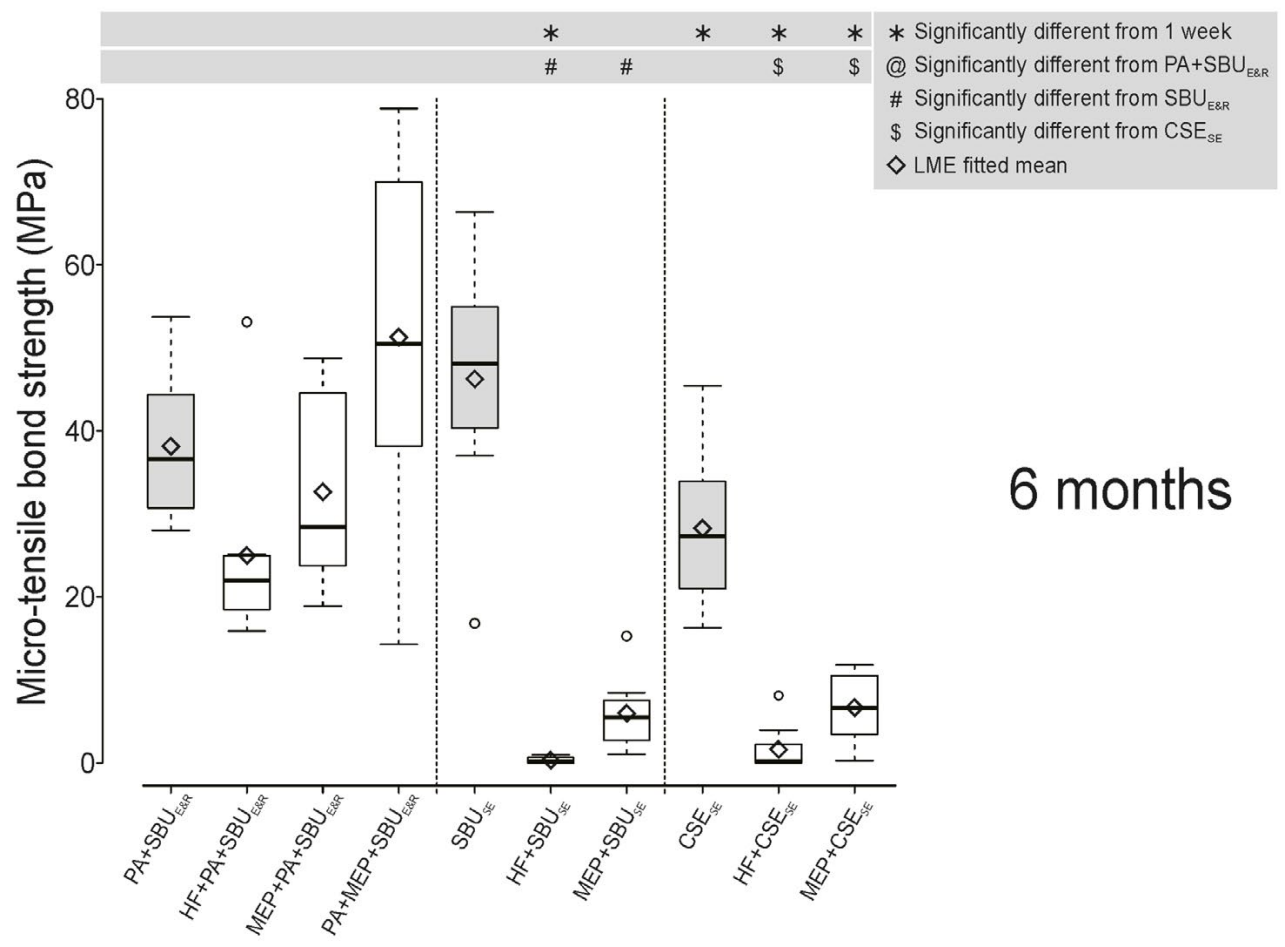
Box plots representing the 6-month micro-tensile bond strength (µTBS in MPa) of the universal adhesive Single Bond Universal (“SBU”; 3M Oral Care/Solventum), applied in etch-and-rinse (E&R) and self-etch (SE) bonding mode, and the considered gold-standard SE adhesive Clearfil SE Bond X (“CSE”; Kuraray Noritake) to dentin non-contaminated (SBU_E&R_, SBU_SE_, and CSE_SE_ controls) and contaminated with either hydrofluoric acid (HF) or the self-etching ceramic primer Monobond Etch & Prime (“MEP”; Ivoclar). The box represents the data spreading between the first and third quartile, with the whiskey denoting minimum and maximum. Black diamonds represent the mean µTBS with the horizontal line referring to the median. Statistically significant differences (P <0.05) are indicated by specific symbols in the figure legend.

The 1-week “immediate” and 6-month “aged” µTBS data are presented in Figures 1 and 2, and numerically detailed in Table 3. Both the highest 1-week and 6-month µTBSs were recorded when dentin was etched with phosphoric acid (PA) followed by “contamination” with the self-etching ceramic primer Monobond Etch & Prime (MEP; Ivoclar) prior to the E&R application of the UA SBU (PA+MEP+SBU_E&R_). In E&R bonding mode, the µTBS of SBU_E&R_ to dentin “contaminated” with any ceramic primer/etchant was not significantly different from the non-contaminated PA+SBU_E&R_ control at 1 week and upon 6-month aging (Figs 1 and 2). However, in SE bonding mode, both the UA SBU_SE_ and the two-step SE adhesive CSE_SE_ bonded significantly less effectively to dentin contaminated with any ceramic primer/etchant, at 1 week and 6 months (HF+SBU_SE_, MEP+SBU_SE_, HF+CSE_SE_, MEP+CSE_SE_). Aging for 6 months did not reduce E&R bonding as compared to the 1-week data, while SE bonding was significantly less effective upon 6-month aging, except for SBU_SE_ and MEP+SBU_SE_. SBU_E&R_ bonding was affected more when dentin was contaminated with MEP before phosphoric acid (PA) etching (MEP+PA+SBU_E&R_) than when dentin was contaminated with MEP after PA etching (PA+MEP+SBU_E&R_).

**Table 3:** Mean 1-week and 6-month micro-tensile bond strength (µTBS in MPa) and failure mode (in %) recorded for the different experimental groups

GROUP	1-WEEK µTBS	Failure analysis^1^	6-MONTH µTBS	Failure analysis^1^
mean (SD^2^)^3^	ptf^4^/n^5^	Adhesive	Mixed	Cohesive	mean (SD)	ptf/n	Adhesive	Mixed	Cohesive
PA+SBU_E&R_	38.9 (6.7)^AB^	0/34	50	12	38	38.1 (9.0)^abc^	0/32	84	3	12
HF+PA+SBU_E&R_	30.0 (5.1)^BC^	0/36	97	0	3	25.0 (11.8)^c^	3/33	85	3	12
MEP+PA+SBU_E&R_	31.0 (5.2)^BD^	0/33	83	3	14	32.6 (11.6)^bc^	0/37	76	8	16
PA+MEP+SBU_E&R_	50.2 (4.6)^A^	0/32	75	9	16	51.3 ( 22.0)^a^	1/32	66	9	25
SBU_SE_	38.0 (9.0)^AB^	0/33	79	6	15	46.2 (14.9)^ab^	0/35	68	6	26
HF+SBU_SE_	11.8 (5.7)^E^	0/32	100	0	0	0.3 (0.4)^d^	28/33	97	0	3
MEP+SBU_SE_	10.0 (2.6)^E^	0/33	100	0	0	6.0 (4.5)^d^	11/32	100	0	0
CSE_SE_	41.0 (8.5)^AB^	0/32	75	12	13	28.3 (9.5)^c^	0/35	74	3	23
HF+CSE_SE_	16.1 (5.9)^CE^	1/32	100	0	0	1.6 (3.0)^d^	22/33	100	0	0
MEP+CSE_SE_	20.0 (7.1)^CDE^	2/32	100	0	0	6.7 (4.1)^d^	8/32	100	0	0
^1^ Adhesive = adhesive failure at the adhesive-dentin interface; Mixed = mixed failure including the adhesive interface along with cohesive failure in resin or dentin; Cohesive = cohesive failure in resin or dentin; ^2^ SD = standard deviation; ^3^ different superscript letters within the column indicate statistically different means (P <0.05); ^4^ ptf = pre-testing failure; ^5^ n = total specimen number.

The failure-mode analysis showed that the specimens failed predominantly “adhesively” in all experimental groups, as displayed in Table 3.

**Fig 3:**
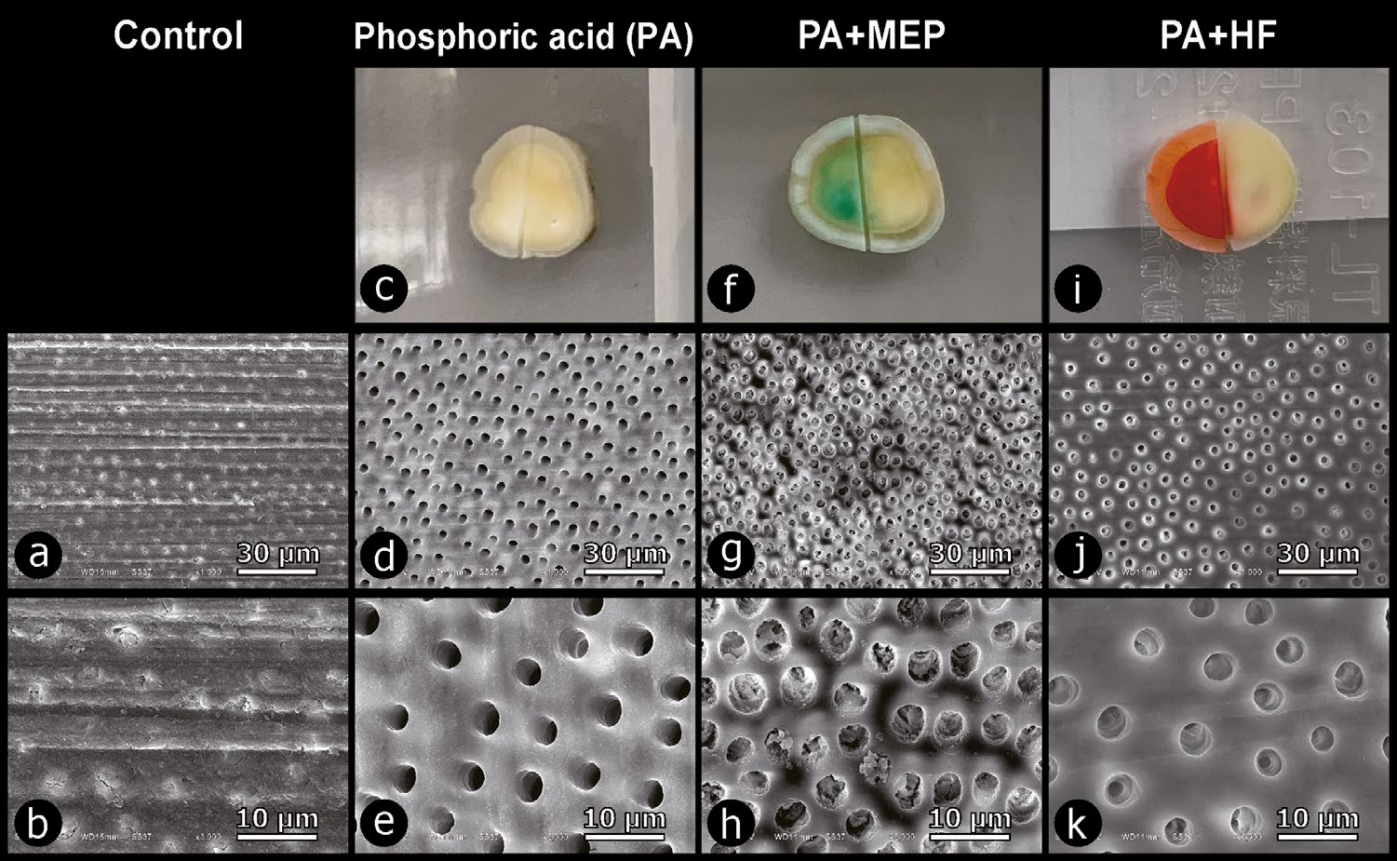
Representative SEM photomicrograph collage of non-primed/etched dentin (non-contaminated control) and dentin contaminated with either phosphoric acid (PA), PA followed by Monobond Etch & Prime (“MEP”; Ivoclar) (PA+MEP), and PA followed by hydrofluoric-acid etching (“HF”; IPS Ceramic Etching Gel, Ivoclar) (PA +HF). Non-primed/etched dentin revealed smear debris-covered dentin with silicon-carbide (SiC) paper scratches and obstructed dentin tubules at 1,000× original magnification in (a) and at 3,000× original magnification in (b). PA-etched dentin in (c) revealed full opening of all dentinal tubules at 1,000× in (d) and 3,000× in (e). Regarding PA+MEP in (f), SEM revealed demineralized dentin, characteristically with partially obstructed dentinal tubules at 1,000× in (g) and 3,000× in (h). Regarding PA+HF in (i), SEM also presented partial obstruction of dentinal tubules at 1,000× in (j) and 3,000× in (k).

**Fig 4:**
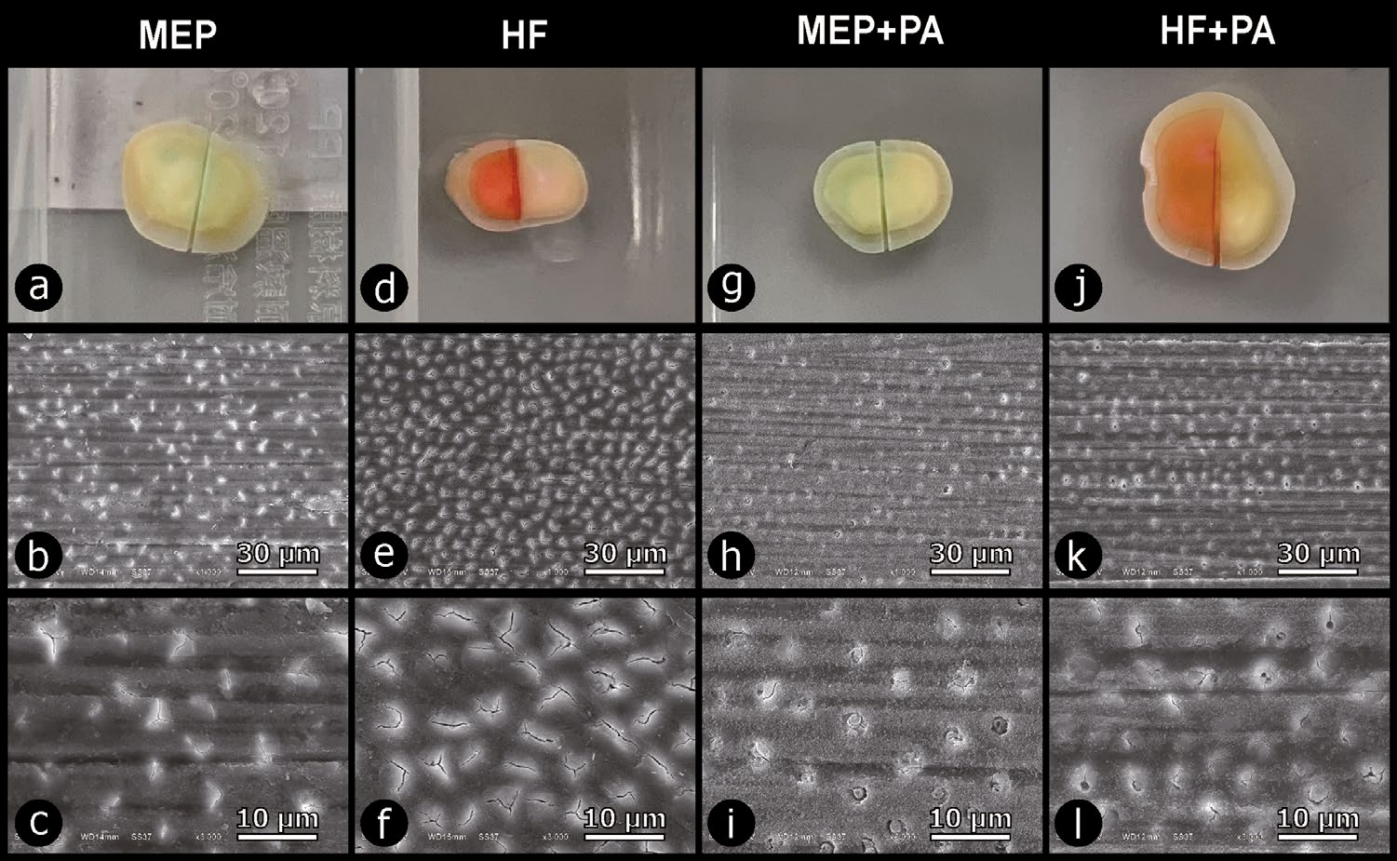
Representative SEM photomicrograph collage of dentin contaminated with Monobond Etch & Prime (“MEP”; Ivoclar) (MEP), hydrofluoric acid etching (“HF”; IPS Ceramic Etching Gel, Ivoclar) (HF), and either MEP followed by phosphoric acid (PA) (MEP+PA) or HF followed by PA (HF+PA). MEP-contaminated dentin in (a), revealed smear debris-covered dentin with silicon-carbide paper scratches and obstructed dentin tubules at 1,000× original magnification in (b) and at 3,000× original magnification in (c). HF-contaminated dentin in (d), revealed smear debris-covered dentin and obstructed dentin tubules at 1,000× in (e) and at 3,000× in (f). Regarding MEP+PA in (g), SEM revealed superficial-demineralized dentin and obstructed dentin tubules at 1,000× in (h) and at 3,000× in (i). Regarding HF+PA in (j), SEM also revealed superficial-demineralised dentin and obstructed dentin tubules at 1,000× in (k) and at 3,000× in (l).

Representative SEM photomicrographs of dentin non-contaminated (control) versus contaminated with MEP or HF following the various experimental protocols are shown in Figures 3 and 4. Digital photos showed green- and red-colored dentin surfaces upon contamination with MEP and HF, respectively. Phosphoric acid (PA) removed the smear layer and opened the dentinal tubule orifices, resulting in completely open tubules (Figs 3d and 3e). MEP and HF reacted with calcium ions on the dentin surface to form insoluble compounds that partly obstructed the dentinal tubules (Fig 4).

The EDS element composition of the differently “contaminated” dentin surfaces is detailed in Table 4. The concentration of phosphorus was lower and that of fluorine higher at dentin contaminated with HF or MEP. Additionally, silicon was detected only in dentin contaminated with MEP.

**Table 4:** Chemical element data of dentin non-primed/etched and contaminated following all experimental groups, as determined by SEM with the energy dispersive X-ray spectroscopy (EDS)

Dentin treatment	Elements (wt%)
Ca	P	F	Si	Ca/P ratio	F/Ca ratio
Non-primed/etched dentin	60.8	32.0	4.1	–	1.9	0.1
Phosphoric acid (PA)	57.9	31.7	7.9	–	1.8	0.1
PA+MEP	47.6	14.7	29.4	5.4	3.2	0.6
PA+HF	53.2	7.9	35.3	–	6.7	0.7
MEP	51.4	22.5	22.0	2.2	2.3	0.4
HF	38.9	8.5	49.8	–	4.6	1.3
MEP+PA	43.0	16.6	37.1	0.9	2.6	0.9
HF+PA	41.1	7.6	48.0	–	5.4	1.2
Ca_10_(PO_4_)_6_(OH)_2_ (theoretical)	40.0	18.5	–	–	2.1	–
CaF_2_ (theoretical)	51.3	–	48.7	–	–	0.9


## DISCUSSION

The bonding effectiveness of adhesives to dentin can be impacted by certain surface treatments, as in this study potential contamination of dentin with ceramic primers/etchants, when employed for ceramic repair, on the bonding receptiveness of adjacently contaminated dentin has been studied. Both null hypotheses were rejected as significant differences in µTBS among the different experimental scenarios and between the 1-week immediate and 6-month aged µTBS were found, particularly for adhesives applied in SE bonding mode.

When the UA Single Bond Universal (3M Oral Care/Solventum) was bonded to dentin in E&R bonding mode (SBU_E&R_), its bonding effectiveness was not significantly affected by any HF or MEP contamination in all the scenario-application sequences investigated. While not significantly different from bonding to non-contaminated dentin (PA+SBU_E&R_), a significantly higher bonding effectiveness was recorded for PA+MEP+SBU_E&R_ as compared to the two other dentin-contamination scenarios HF+PA+SBU_E&R_ and MEP+PA+SBU_E&R_. This means that when repairing ceramic restorations, adjacent dentin is best first etched with phosphoric acid prior to the application of MEP onto the ceramic. Unfortunately, a PA+HF+SBU_E&R_ scenario was not included in the study design. Nevertheless SEM revealed relatively similar surface morphology upon PA+MEP and PA+HF, by which most likely a similar better bonding performance can also be expected for PA+HF+SBU_E&R_ in line of that measured for PA+MEP+SBU_E&R_.

Morphologically, the MEP-, HF-, MEP+PA-, and the HF+PA-contaminated surfaces appear less bonding receptive, clearly showing residual smear-layer scratches and severely obstructed dentin tubules, as imaged by SEM. These unfavorable surface morphologies may explain the lower 1-week and 6-month µTBS measured for HF+PA+SBU_E&R_ and MEP+PA+SBU_E&R_, though still not significantly different from the PA+SBU_E&R_ control µTBS. When dentin was etched with PA solely or additionally contaminated with MEP (PA+MEP) and HF (PA+HF), smear-layer scratches were no longer observed, indicating that the smear layer was effectively removed by PA, while MEP and HF clearly less obstructed the dentinal tubules, most likely having resulted in the higher bonding performance recorded for PA+MEP+SBU_E&R_.

When the UA Single Bond Universal (3M Oral Care/Solventum) was bonded to dentin in SE bonding mode without PA (SBU_SE_), prior contamination with HF (HF+SBU_SE_) and MEP (MEP+SBU_SE_) severely/significantly affected the bonding receptiveness of dentin, indicating that the interaction of the relatively mild 10-MDP-based UA was insufficient to deal with the unfavorable HF/MEP-contaminated dentin-surface morphology observed by SEM. Even the separately applied 10-MDP-based primer as part of the considered gold-standard SE adhesive Clearfil SE Bond X (Kuraray Noritake) appeared incapable of restoring the bonding receptiveness of dentin, as significantly lower µTBS was also recorded for HF+CSE_SE_ and MEP+CSE_SE_.

MEP is a self-etching ceramic primer containing the HF derivative tetrabutylammonium dihydrogen trifluoride along with the metacrylate silane 3-(trimethoxysilyl)propyl methacrylate. This simultaneously etches and primes (glass-)ceramic for adhesive luting purposes and hereby offers a less aggressive surface (pre)treatment option, which certainly is safer to use than HF when repairing ceramic restorations intraorally. When the dentin surface is contaminated with MEP, fluoride ions from this substance react with calcium ions abundantly available at dentin to form insoluble ionic fluorocalcium salt (CaF_2_) that precipitates on the dentin surface and obstructs the dentinal tubules. The water solubility of CaF_2_ is low, even at a low pH level.^15^ This causes CaF_2_ to precipitate, thereby impeding the infiltration of adhesive components into dentin. Dentinal tubule obstruction induced by MEP was confirmed by SEM (Figs 3 and 4), while EDS (Table 4) demonstrated an increase in fluorine on MEP-contaminated dentin. Like MEP, HF resulted in similar dentin-tubule closure. SEM revealed that even etching MEP- and HF-contaminated dentin with PA did not (fully) open the dentin tubules, indicating how severely dentin was contaminated/affected by MEP and HF. In other words, PA was not capable of sufficiently dissolving the CaF_2_ precipitation.

Such CaF_2_ precipitates were mentioned before to reduce the bonding effectiveness of adhesives due to impeded adhesive infiltration into dentin.^12^ Previous studies have indeed shown that etching dentin with solely PA resulted in significantly higher µTBS than when dentin was etched with 3% and 9.6% HF.^12,20^ TEM interfacial characterization revealed that dentin etched/contaminated with HF causes the exposed collagen to collapse, thereby inhibiting adequate hybridization.^12^ Besides, thinner hybrid layers at HF-etched/contaminated dentin were observed versus solely PA-etched dentin; collagen fibrils appeared severely affected by HF, which even resulted in interfacial de-bonding.^12^ Szep et al (2000) and Pioch et al (2003) reported before that HF enriched the dentin surface in fluoride, and claimed that this makes the surface more resistant to acid and hampers the etching effect of PA.^18,24^ They also explained that the CaF_2_ precipitates formed by HF are basically insoluble and block the subsequently applied PA. As a result, interfacial gap formation was observed using TEM by Loomans et al in 2010.^12^

Interestingly, Loomans et al (2010) investigated both scenarios of etching dentin first with 3% and 9.6% HF, followed by post-etching with PA, as well as vice versa, etching dentin first with PA, followed by etching with HF.^12^ Both scenarios resulted in significantly reduced µTBS of the considered gold-standard E&R adhesive Optibond FL (Kerr, Orange, CA, USA), as compared to its µTBS to dentin solely etched with PA. Their finding that etching dentin first with HF followed by etching with PA reduced µTBS aligns with the finding in this study that HF+PA+SBU_E&R_ reduced µTBS, though not significantly in this study. As SEM revealed similar surface morphology upon PA+MEP and PA+HF, it was mentioned above that most likely a similar better bonding performance could be expected for PA+HF+SBU_E&R_, which was not measured in this study but could be extrapolated based on the data measured for PA+MEP+SBU_E&R_. The study of Loomans et al (2010) however reported the contrary that prior etching with PA followed by HF etching was as detrimental for adhesion, by which next research definitely should also investigate the PA+HF+SBU_E&R_ scenario.^12^ The paper of Loomans et al (2010) was clear and definitive that HF on dentin should be avoided. This indicates that the bond-promoting effect of MEP upon PA etching cannot be extrapolated to HF upon PA etching.

While confirming the previous study of Loomans et al (2010),^12^ the reduced µTBS of the UA Single Bond Universal (SBU; 3M Oral Care/Solventum) upon prior HF etching (HF+PA+SBU_E&R_ in this study) was not significant upon comparison with the non-contaminated PA+SBU_E&R_ control. Most likely, this should be related to the use of different adhesives in both studies. SBU contains the 10-MDP functional monomer in addition to the copolymer of acrylic and itaconic acid, the latter before being referred to by 3M as the Vitrebond polyalkenoic-acid copolymer. Both 10-MDP and the copolymer in SBU have been demonstrated to chemically interact with dentinal hydroxyapatite (HAp), which is in line with the adhesion-decalcification (AD) concept formulated before.^26,27^ While the dedicated E&R adhesive Optibond FL (Kerr) does not contain 10-MDP, the additional chemical interaction potential of SBU with dentinal HAp may hence explain why the nevertheless reduced µTBS was not significantly lower than that of the PA+SBU_E&R_ control. Additionally, the previous study used stannous fluoride on the dentin surface, which reacts with HAp and produces products such as stannous fluorophosphate (Sn_3_F_3_PO_4_), calcium fluoride (CaF_2_), and calcium hydroxide (CaOH_2_).^3,14^ The purpose was to enhance the chemical interaction of polycarboxylate cement with dentin.^5,14^ Therefore, the presence of 10-MDP and polyalkenoic acid in SBU, both having chemical interaction potential, may have reduced the adverse effect of the precipitated CaF_2_.

The finding that contamination of dentin with HF or MEP before applying the adhesive negatively impacted bonding effectiveness, corresponded also to the more recent study.^10^ They investigated the influence of surface contamination via airborne-particle abrading, silica coating, hydrofluoric-acid etching, and a self-etching ceramic primer on the shear bond strength (SBS) of the UA Adhese Universal (Ivoclar) to bovine enamel and dentin when applied in E&R and SE bonding modes. In E&R bonding mode, post-etching contamination by sandblasting and silica coating significantly reduced SBS to enamel and dentin, while hydrofluoric acid impaired enamel bonding. In the SE bonding mode, all contaminants significantly lowered SBS to enamel and dentin, except for the universal primer (Monobond Plus, Ivoclar).^10^ As mentioned earlier, the rather insoluble CaF_2_ blockage caused by MEP (MEP+SBU_SE_, MEP+CSE_SE_) and HF (HF+SBU_SE_, HF+CSE_SE_) on the dentin surface, along with CaF_2_-salt precipitation obstructing the dentinal tubules, must have severely hindered the interaction of SBU applied in SE bonding mode (SBU_SE_), similar to that of CSE_SE_.

Further, EDS revealed an increase in fluorine in dentin contaminated with MEP and HF, resulting in the recorded higher F/Ca ratios (Table 4), also in agreement with previous research.^18^ MEP contains bis(triethoxysilyl)ethane (1–2.5 wt%) and 3-(trimethoxysilyl)propyl methacrylate, by which silicon (Si) was identified on MEP-contaminated dentin. Interestingly, MEP+PA-contaminated dentin showed a reduction in silicon compared to solely MEP-contaminated dentin. Self-evidently, this reduction must be attributed to silicon removal by PA.

Overall, when intraorally repairing ceramic restorations, contamination of dentin with MEP and HF must be avoided, definitely when the adhesive is applied in SE bonding mode, but also in E&R bonding mode. This study showed that bonding effectiveness to dentin was solely not affected when dentin was etched first with PA and the MEP contamination occurred following PA etching prior to the adhesive application. Therefore, in clinical situations where the dentin surface may be contaminated with HF or MEP, an E&R bonding mode using a universal adhesive is recommended.

Furthermore, the residual color of the HF- and MEP-contaminated dentin surface remaining despite water rinsing could potentially have unfavorable esthetic effects.

The findings presented in this study are of great significance to clinical practice, where the use of adhesives in the repair of (glass-)ceramics is common. It emphasizes the importance of ensuring that dentin surfaces are free from contamination to ensure optimal adhesion.

The present study provides valuable insights into the decontamination efficacy of specific adhesives. However, several study limitations should be acknowledged to contextualize our findings. A first limitation is the number/scope of adhesives tested. Though market-representative, our study exclusively investigated one universal adhesive (SBU) and one two-step self-etch adhesive (CSE), both containing 10-MDP as functional monomer, with the latter adhesive being considered as a gold-standard self-etch (SE) adhesive. While it is plausible that other adhesives may exhibit similar behavior depending on their bonding strategy, care should be taken when generalizing these findings to the broader categories of universal and self-etch adhesives. Future research could aim to include a more diverse range of adhesives to validate and expand our understanding of their performance.

Another notable limitation is the use of non-carious human third molars. This model was chosen to ensure a standardized, controlled experimental environment. However, it does not fully replicate the complexity of clinical conditions. In practice, dentists often encounter caries-affected or sclerotic dentin,^9^ which may respond differently to adhesives due to variations in chemical and morphological properties. Therefore, while our results are robust for sound dentin, they may not be directly applicable to situations involving compromised dentin. Future studies could investigate the effectiveness of these decontamination protocols on a variety of clinically relevant dentin substrates.

## CONCLUSION

Particular (glass-)ceramic primers/etchants, such as Monobond Etch & Prime (“MEP” in this study; Ivoclar) and hydrofluoric acid (“HF” in this study; IPS Ceramic Etching gel, Ivoclar), can negatively affect μTBS to dentin. With potential MEP/HF contamination, a UA should definitely not be applied in SE bonding mode. MEP/HF contamination prior to phosphoric acid etching (“PA” in this study) also reduced the E&R bonding performance. The sequence of contamination, however, has an impact on the adhesion performance when using an E&R bonding mode. Interestingly, contamination with MEP after PA etching resulted in significantly higher μTBS compared to MEP/HF contamination prior to PA etching.

### Acknowledgments

This study was partially supported by the Coordinating Center for Thai Government Science and Technology Scholarship Students (CSTS) and the National Science and Technology Development Agency (NSTDA) [grant number JRA-CO-2564-14768-TH].

### Clinical Relevance

Contamination of dentin with ceramic primers/etchants (Monobond Etch & Prime, Ivoclar [“MEP”], or hydrofluoric acid [“HF”]) can negatively affect dentin-bonding receptiveness. Self-etch application of universal adhesives is not advised with MEP/HF contamination. While MEP/HF contamination prior to phosphoric acid etching (PA) weakens etch-and-rinse bonding, MEP contamination after PA unexpectedly results in higher μTBS compared to pre-priming/etching contamination.
